# Successful Use of Calcium Chloride in Acute Calcium Channel Blocker Overdose With Shock: A Case Report

**DOI:** 10.7759/cureus.80320

**Published:** 2025-03-10

**Authors:** Shunsuke Nakamura, Natsuyo Shinohara

**Affiliations:** 1 Department of Emergency Medicine, Okayama Rosai Hospital, Okayama, JPN

**Keywords:** calcium channel blockers (ccbs), calcium chloride, overdose, shock, toxicity

## Abstract

Calcium channel blockers (CCBs) are widely used to treat hypertension and tachyarrhythmias. However, an overdose can lead to severe toxicity, resulting in bradycardia, hypotension, and shock. Standard treatments, including calcium gluconate and catecholamines, may be ineffective in severe cases. This study reports a 60-year-old female with hypotensive shock following an intentional overdose of amlodipine. Despite the initial treatment with calcium gluconate, glucagon, and catecholamines, hemodynamic instability persisted. However, the intravenous administration of calcium chloride resulted in rapid hemodynamic improvement, allowing eventual recovery. This case highlights the potential of calcium chloride as a first-line antidote for severe CCBs overdose refractory to conventional treatments.

## Introduction

Calcium channel blockers (CCBs) are commonly prescribed for hypertension and dysrhythmias, but overdose can cause life-threatening toxicity [1]. The underlying toxicity mechanism involves the inhibition of L-type calcium channels, causing reduced intracellular calcium, impaired myocardial contractility, and vasodilation, ultimately resulting in hypotension and shock [2]. Recent reports have indicated the efficacy of calcium chloride in the treatment of acute drug poisoning caused by CCBs. They exert toxicity by binding to the α1 subunit of the L-type Ca^2+^ channel and inhibiting Ca2+ influx. This results in decreased myocardial contractility, peripheral vascular resistance, and suppression of insulin secretion, leading to acute circulatory failure and metabolic acidosis [3-5].

Conventional treatment includes intravenous fluids, calcium supplementation, glucagon, and high-dose catecholamines; however, these therapies may prove ineffective in severe cases. Recent studies have shown that ionized calcium is directly available via calcium chloride, making it an efficient agent than calcium gluconate in managing acute toxicity [2]. However, calcium chloride is typically administered via a central venous catheter due to its vascular irritancy. Its higher threshold for use compared to calcium gluconate is a disadvantage. Additionally, high-dose insulin euglycemia therapy (HIET) has emerged as a promising adjunctive therapy for patients with CCB overdose who do not respond to conventional treatments [6].

This report presents a case of acute amlodipine overdose in which calcium chloride successfully stabilized circulation after standard treatments failed.

## Case presentation

**Figure 1:**
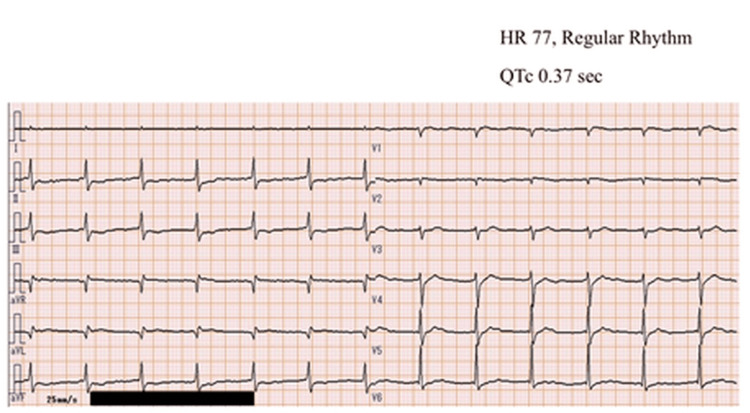
12-lead electrocardiogram at presentation. The electrocardiogram on admission shows sinus rhythm with QTc 0.37 seconds.

A 60-year-old female with a medical history of hypertension, left cerebral hemorrhage, and prior emergency transport for a suicide attempt ingested 390 mg of amlodipine in a suicide attempt. The following morning, the patient was presented to a local clinic with altered consciousness and hypotension. Thereafter, she was transferred to our emergency department. The preliminary vitals were recorded as follows: Glasgow Coma Scale (GCS): E3V4M5, pulse: 70 bpm, sinus rhythm, respiration rate: 20 breaths/min, SpO_2_: 98% on room air, temperature: 35.9°C, echocardiography: diffuse hypodynamic state. Blood pressure could not be measured, whereas 12-lead electrocardiography (ECG) showed no QT prolongation, with a QTc of 0.37 seconds (Figure 1).

As demonstrated in Table 1, the initial laboratory data indicated the presence of severe circulatory failure and liver damage.

**Table 1:** The initial laboratory data. WBC: white blood cell, RBC: red blood cell, Hb: hemoglobin, Ht: hematocrit, PLT: platelet, AST: aspartate aminotransferase, ALT: alanine aminotransferase, LD: lactate dehydrogenase, UN: urea nitrogen, Cre: creatinine, T.Bil: total bilirubin, CRP: C-reactive protein, Alb: albumin, PT: prothrombin time, INR: international normalized ratio, APTT: activated partial thromboplastin time, Fibg: fibrinogen, Lac: lactate, BE: base excess, Glu: glucose, FiO_2_: fraction of inhaled oxygen; PaO_2_: partial pressure of oxygen ; PaCO_2_: partial pressure of carbon dioxide; HCO_3_-: bicarbonate ion.

Test	Result	Units	Reference values
White blood cell	11240	/μL	3300-8600
Red blood cell	4.48*10^6^	/μL	4.35-5.55*10^6^
Hemoglobin	12.9	g/dL	13.7-16.8
Hematocrit	39.3	%	40.7-50.1
Platelet	173*10^3^	/μL	155-348*10^3^
Aspartate aminotransferase	869	U/L	13-30
Alanine aminotransferase	551	U/L	Oct-42
Lactate dehydrogenase	801	U/L	124-222
Urea nitrogen	31.9	U/L	20-Aug
Creatinine	1.71	mg/dL	0.65-1.07
Total bilirubin	0.8	mg/dL	0.4-1.5
C-reactive protein	0.1	mg/dL	<0.14
Sodium	140	mmol/L	138-145
Potassium	3.6	mmol/L	3.6-4.8
Chlorine	102	mmol/L	101-108
Albumin	3.5	g/dL	4.1-5.1
PT(%)	134	%	80-120
PT-international normalized ratio	0.86		0.9-1.1
Activated partial thromboplastin time	< 23.0	second	25-40
Fibrinogen	333	mg/dL	200-400
D-dimer	1.1	μg/mL	<1.0
pH	7.33		7.35-7.45
FiO_2_	0.21		-
PaO_2_	113	mmHg	80-100
PaCO_2_	30	mmHg	35-45
HCO_3_^-^	15.8	mmol/L	22-26
Lac	9.6	mmol/L	0.5-2.2
BE	-8.8	mmol/L	-2 to +2
Glu	350	mg/dL	70-140

**Figure 2:**
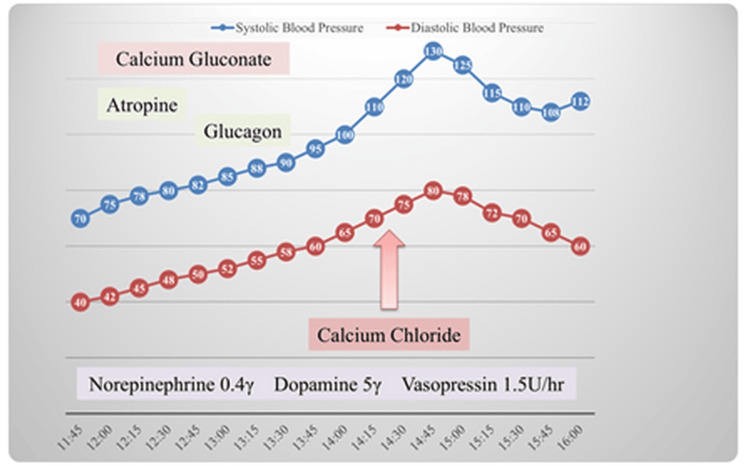
The course after admission. Hemodynamic response to calcium chloride administration.

Continuous intravenous infusions of norepinephrine (0.4 μg/kg/min), dopamine (5 μg/kg/min), vasopressin (1.5 units/hour), and other medications such as atropine, calcium gluconate, and glucagon were administered intravenously as required. However, the systolic blood pressure remained at 70 mmHg. Owing to persistent hypotension, the patient was initially administered intravenous fluids, calcium gluconate, glucagon, and catecholamines, but these did not achieve the desired hemodynamic stability. Therefore, considering the severity of the circulatory failure, intravenous calcium chloride (2%) administration was initiated at 14:30 (the administration of the 20 ml solution was conducted intravenously over a period of approximately five minutes), which resulted in a significant improvement in blood pressure and perfusion (Figure 2).

**Figure 3:**
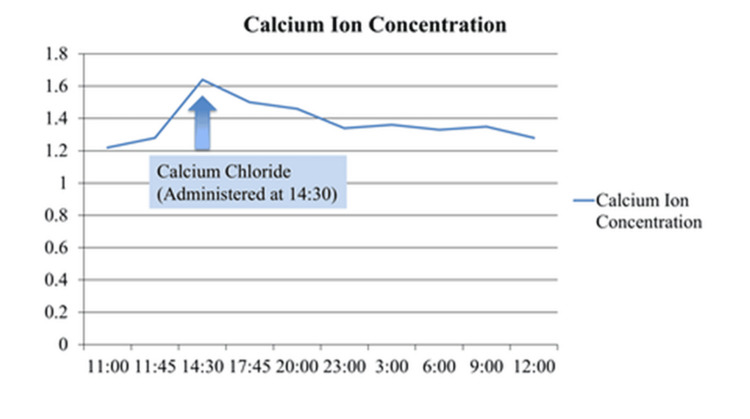
The blood calcium ion concentration. This figure shows the changes in blood calcium ion concentration (mmol/L) over time, highlighting a peak following the administration of calcium chloride at 14:30.

Immediately after the aforementioned treatment, blood calcium ion concentrations increased marginally (Figure 3).

The patient’s hospital course is outlined below. On hospital day (HD) 2, the patient developed paroxysmal atrial fibrillation, which was managed with intermittent calcium chloride administration and electrolyte correction. By HD 5, the patient was transferred from the intensive care unit and finally discharged after the psychiatric evaluation on HD 7. Forensic procedures have not been conducted.

## Discussion

Recent reports have indicated the efficacy of calcium chloride in the treatment of acute drug poisoning caused by CCBs. They exert toxicity by binding to the α1 subunit of the L-type Ca^2+^ channel and inhibiting Ca^2+^ influx. This results in decreased myocardial contractility, peripheral vascular resistance, and suppression of insulin secretion, leading to acute circulatory failure and metabolic acidosis [3-5].

While calcium gluconate is frequently employed in the treatment of CCB poisoning, its efficacy is contingent on the time elapsed, as it requires hepatic metabolism to increase the ionized calcium concentration in the blood. Conversely, calcium chloride does not involve hepatic metabolism and can expeditiously augment the ionized calcium concentration in blood. Consequently, calcium chloride has been postulated to be more effective in cases of acute circulatory failure; despite the administration of calcium gluconate, glucagon, and high-dose catecholamines, these interventions were insufficient [2,7-9].

Hence, this case highlighted the potential benefits of calcium chloride as a primary counter-agent for CCB poisoning, especially in case of acute circulatory collapse. In critical situations, such as the present case, where the patient exhibits severe circulatory or hepatic failure, it is preferable to administer the drug either by promptly securing a central venous catheter or by administering it slowly and carefully through a peripheral vein.

## Conclusions

In calcium antagonist poisoning where the patient does not respond to calcium gluconate or other treatments, calcium chloride administration is an effective option. However, in acute circulatory failure, such as the aforementioned case, calcium chloride is believed to be the optimal choice because of its rapid increase in ionized calcium concentration in the blood without the need for hepatic metabolism.
